# Post-Covid-19 condition (Long Covid) in children and young people 12 months after infection or reinfection with the Omicron variant: a prospective observational study

**DOI:** 10.1038/s41598-024-60372-4

**Published:** 2024-04-30

**Authors:** Snehal M. Pinto Pereira, Manjula D. Nugawela, Terence Stephenson, Paul Foret-Bruno, Emma Dalrymple, Laila Xu, Elizabeth Whittaker, Isobel Heyman, Tamsin Ford, Terry Segal, Trudie Chalder, Shamez N. Ladhani, Anna A. Mensah, Kelsey McOwat, Ruth Simmons, CLoCk Consortium, Marta Buszewicz, Esther Crawley, Shruti Garg, Dougal Hargreaves, Anthony Harnden, Michael Levin, Vanessa Poustie, Malcolm Semple, Kishan Sharma, Bianca De Stavola, Olivia Swann, Roz Shafran

**Affiliations:** 1 Division of Surgery and Interventional Science, Faculty of Medical Sciences, University College London, Gower Street, London, WC1E 6BT UK; 2 UCL Great Ormond Street Institute of Child Health, University College London, 30 Guilford Street, London, WC1N 1EH UK; 3 Institut de Psychologie, Université Lumière Lyon 2, 18 Quai Claude Bernard, 69365 Lyon Cedex 07, France; 4 Section of Paediatric Infectious Diseases, Imperial College London, London, UK; 5 Paediatric Infectious Diseases, Imperial College Healthcare NHS Trust, London, UK; 6 Department of Psychiatry, University of Cambridge, Hershel Smith Building Cambridge Biomedical Campus, Cambridge, CB2 0SZ UK; 7 University College London Hospitals NHS Foundation Trust, London, UK; 8 Department of Psychological Medicine, Institute of Psychiatry, Psychology and Neuroscience, King’s College London, De’Crespigny Park, London, SE5 8AF UK; 9 Immunisations and Vaccine Preventable Diseases, UK Health Security Agency, 61 Colindale Avenue, London, NW9 5EQ UK; 10 University College London, London, UK; 11 University of Bristol, Bristol, UK; 12 University of Manchester, Manchester, UK; 13 Imperial College London, London, UK; 14 Oxford University, Oxford, UK; 15 University of Liverpool, Liverpool, UK; 16 Manchester University NHS Foundation Trust, Manchester, UK; 17 University of Edinburgh, Edinburgh, UK

**Keywords:** Post Covid-19 condition, Omicron, Long Covid, Young people, Prospective, Signs and symptoms, Diseases

## Abstract

Our previous study in children and young people (CYP) at 3- and 6-months post-infection showed that 12–16% of those infected with the Omicron (B.1.1.529) variant of SARS-CoV-2 met the research definition of Long Covid, with no differences between first-positive and reinfected CYP. The primary objective of the current study is to explore the impact of the Omicron variant of SARS-CoV-2 infection on young people 12 months post infection. 345 CYP aged 11–17 years with a first laboratory-confirmed infection with the Omicron variant and 360 CYP reinfected with the Omicron variant completed an online questionnaire assessing demographics, symptoms, and their impact shortly after testing and again at 3-, 6-and 12-months post-testing. Vaccination status was determined from information held at UKHSA. Comparisons between groups were made using chi-squared, Mann–Whitney U, and Kruskal–Wallis tests. The most common symptoms in first-positive and reinfected CYP 12-months post-testing were tiredness (35.7 and 33.6% respectively) and sleeping difficulties (27.5 and 28.3% respectively). Symptom profiles, severity and impact were similar in the two infection status groups. Overall, by 12-months, 17.4% of first-positives and 21.9% of reinfected CYP fulfilled the research consensus Long Covid definition (*p* = 0.13). 12-months post Omicron infection, there is little difference between first-positive and reinfected CYP with respect to symptom profiles and impact. Clinicians may not therefore need to consider number of infections and type of variant when developing treatment plans. Further studies are needed to assess causality of reported symptoms up to 12-months after SARS-CoV-2 infection.

## Introduction

Post-Covid-19 condition (PCC), also known as Long Covid, is characterised by impairing symptoms that persist, but can relapse and remit, at least 12 weeks after SARS-CoV-2 infection^1^. PCC has been described as threatening individuals, populations, and economies^2^. In the UK, national surveys in February 2023 suggest 4% of adults and 1% of children and young people (CYP) aged 2-to-15 years old, report Long Covid, characterised by difficulties with fatigue, concentration, muscle aches, and shortness of breath^3^. Many of these people may have now been living with the symptom complex for several years. For CYP specifically, this may have a detrimental impact on their development and education.

Systematic reviews have identified over two-hundred symptoms associated with PCC in CYP, including headaches, shortness of breath, fatigue and cough^4,5^. However, research into the long-term impact of SARS-CoV-2 has been challenging for a number of reasons. Definitions of PCC in CYP have only relatively recently been proposed^1,6^ and only one-third of studies utilise a recognised definition^7^. Almost 82.0% of 5–11-year-olds and 99.3% of 12–18-year-olds had SARS-CoV-2 antibodies by June 2022 in the UK, which means that it is no longer possible to have a comparator group of test-negative CYP^8^. Some CYP are asymptomatic and, coupled with the removal of mandatory testing and reporting, it is difficult to establish how many infections a young person may have experienced since the start of the pandemic or the impact of repeated infection. The introduction of COVID-19 vaccination is a further important factor that needs to be taken into consideration when examining the long-term impact of SARS-CoV-2 infection.

Despite these challenges, it is critically important to try to understand the long-term impact of SARS-CoV-2 infection, particularly in CYP for whom the pandemic and sequelae of infection, influence many aspects of their lives including, for example, detrimental effects on learning^9^. The CLoCk study is the largest national, matched longitudinal study of CYP in England, where CYP self-report post-Covid health problems after a laboratory-confirmed SARS-CoV-2 infection between September 2020 and March 2021^10^. The findings from CLoCk at 3-, 6- and 12-months post-testing have been published elsewhere^11–14^. Collectively these publications provide data on the proportion of CYP meeting the Delphi research definition of Long Covid^1^ at various time-points post-infection, with 24–28% of test-positive and 17–21% of test-negative CYP meeting this definition when data are examined cross-sectionally^12,14,15^. The research also demonstrates the importance of examining data longitudinally as the findings showed that whilst overall prevalence of 9 of the most common 11 symptoms declined by 12-months, many CYP were reporting key symptoms such as shortness of breath and tiredness for the first time at 6- or 12-months post-testing^12^.

Most of the CLoCk cohort are likely to have been infected with wild type SARS-CoV-2 (between September and December 2020) and the Alpha (B.1.1.7) variant (between January and March 2021)^16^. However, the Omicron (B.1.1.529) variant of SARS-CoV-2 emerged in November 2021 and spread rapidly, with more cases during December 2021 and March 2022 than all previous cases combined^17^. The question of the long-term impact of infection with the Omicron variant could not be addressed by the original CLoCk study but, CLoCk’s established methodology enabled a smaller sub-study to be rapidly set up to collect data at 0-, 3-, 6- and 12-months post-infection in (a) test-positive CYP infected for the first time during the period when Omicron was dominant, (b) CYP who had more than one infection, with reinfection occurring during the period when Omicron was dominant and (c) matched test-negative CYP. The original CLoCk study was unable to collect contemporaneous information from CYP at the time of infection but this sub-study was able to do so, thereby minimising recall bias. The Omicron sub-study showed that 12–16% of those infected with Omicron met the research definition of Long Covid at 3- and 6-months post-infection with no evidence of difference between first-positives and reinfected^18^.

Data are now available to examine the impact of infection with the Omicron variant in this cohort at 12-months post-infection. For reasons explained above, we no longer consider the test-negative group of CYP as controls. Instead, this analysis aims to provide the first report of the long-term (12-month) follow-up of the sub-sample who had been infected with Omicron, considering those that were infected for the first time when Omicron was the dominant strain and those that were reinfected during the Omicron period. Based on data from the larger CLoCk study and our previous report of the Omicron sub-study^12,18^, the following hypotheses were made: that 12-months post-infection (i) the most common symptoms CYP will be experiencing will be headaches, poor sleep, shortness of breath and tiredness, (ii) the overall prevalence of symptoms will be lower compared to baseline, 3- and 6-months post-infection, (iii) there will be no difference in symptoms between the reinfected group and first-positive group, and (iv) symptoms will not differ by vaccination status. In addition, we hypothesise that longitudinally: (v) the within-individual prevalence of symptoms in the reinfected and first-positives (who report the symptom at baseline) will decline by 12-months, and likewise (vi) the within-individual prevalence of symptoms in the reinfected and first-positives who report the symptom for the first time at 3-/6-months will decline by 12 months.

## Method

**Figure 1:**
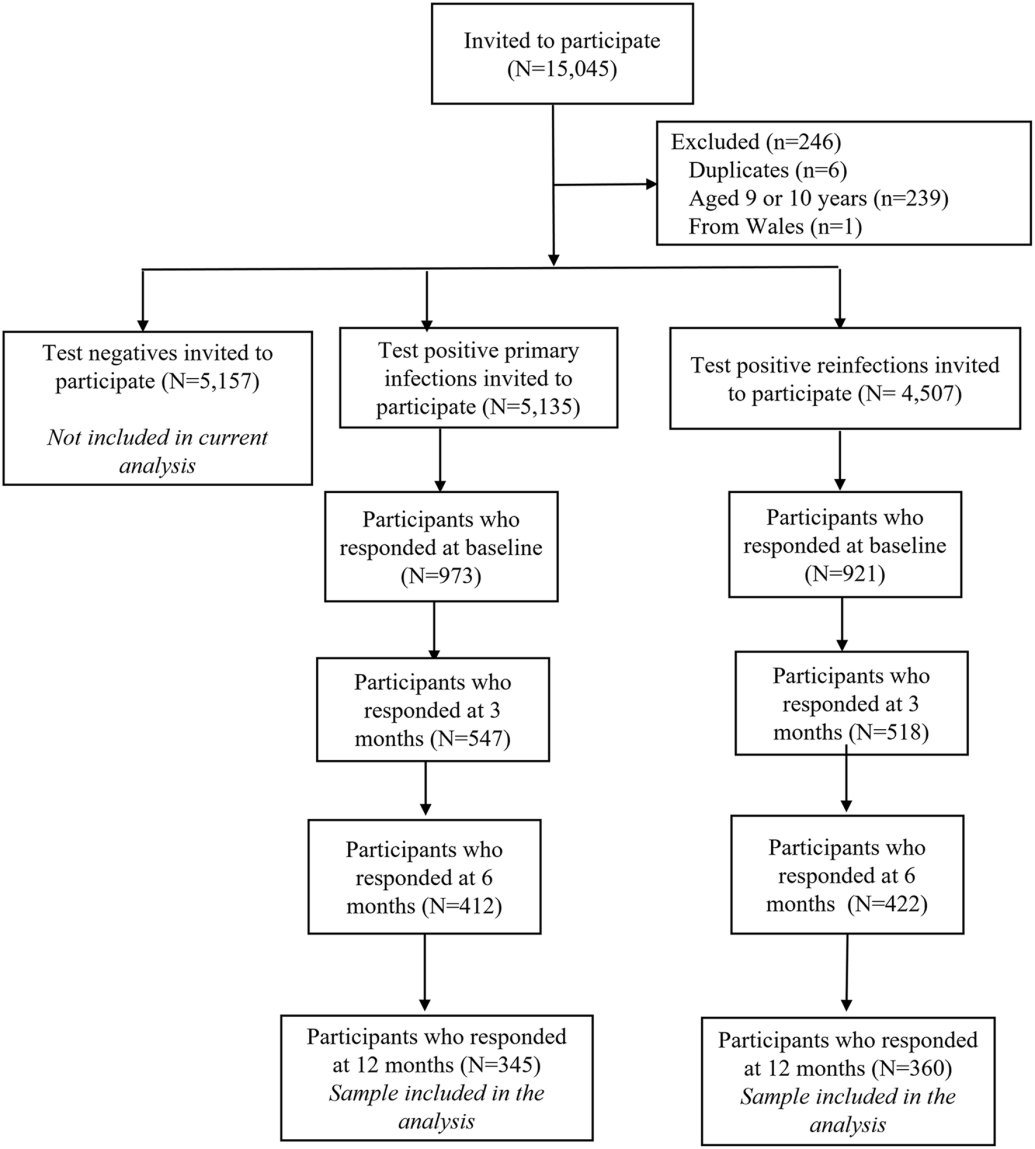
Participant flow diagram for the CLoCk Omicron sub-study.

CLoCk study methodology and this sub-study have both been described elsewhere^10,18^. Briefly, for the Omicron sub-study, 15,045 CYP aged 11–17 who had a SARS-CoV-2 PCR test in January 2022 were invited by mail to participate. The first-positives were matched at study invitation to test-negatives by age (at last birthday), sex and geographical area (based on lower super output area) using the national SARS-CoV-2 testing database held at the UK Health Security Agency (UKHSA); all reinfected CYP were invited (Fig. 1). Consenting CYP filled in an online questionnaire shortly after testing (i.e., at 0-months post-testing) and again at 3-, 6-and 12-months post-testing. The CYP were able to ask their parents for assistance if required e.g., due to neurodiversity. The questionnaires included demographics, elements of the International Severe Acute Respiratory and Emerging Infection Consortium (ISARIC) questionnaire, 28 symptoms (e.g., shortness of breath, tiredness, brain fog), as well as validated scales including the EQ-5D-Y^19^ (which measures health-related quality of life). The Delphi research definition of Long Covid in CYP^1^ was operationalised at the time of questionnaire completion 3-, 6-and 12-months post-testing as experiencing ≥ 1 symptom AND problems with mobility, self-care, doing usual activities or having pain/discomfort or feeling very worried/sad, based on the EQ-5D-Y scale. CYP meeting this operationalised research definition were classified as having Long Covid. Vaccination status (dosage: 0, 1, 2, 3+) was determined from information held at UKHSA; when information was missing (n = 11), self-reported information from the 12-month post-testing questionnaire was used.

### Statistical methods

To assess representativeness of our analytical sample (see Fig. 1), we compared their demographic characteristics (sex, age, region of residence, and Index of Multiple Deprivation (IMD)) to the target population (i.e., all those invited to take part in the Omicron sub-study) by infection status. To determine the most common symptoms CYP experience at 12-months (objective i) and whether the overall prevalence of symptoms was lower compared to previous data collection sweeps (objective ii), we describe the prevalence of individual symptoms over the study period (i.e., at 0-, 3-, 6- and 12-months). To determine if symptom profiles were similar in the reinfected and first-positive groups, 12-months post-testing (objective iii), we describe the total number of symptoms reported (0, 1, 2, 3, 4 and 5+), prevalence of individual symptoms, self-rated health, symptom impact and severity 12-months post-testing by infection status. To determine if symptoms differ by vaccination status (objective iv), we describe study participant characteristics on symptoms and validated scales, 12-months post-testing by infection status and vaccination status at 12-months. To determine if the prevalence of symptoms in the reinfected and first-positives (who report the symptom at baseline) declined by 12-months, and likewise if the prevalence of symptoms in the reinfected and first-positives (who report the symptom for the first time at 3-/6-months) declined by 12-months (objectives v and vi), we generated stacked bar charts showing the distribution of (a) individual symptoms at 0-, 3-, 6-months and 12-months and (b) Long Covid at 3-, 6-and 12-months by the two infection-status groups, indicating when the symptom was first reported/ the Long Covid definition was first met. Chi-squared tests were used to examine associations between infection-status groups for binary/categorical variables; continuous variables (self-rated health, symptom severity and symptom impact) were examined using Mann–Whitney U or Kruskal–Wallis tests.

The study was approved by Yorkshire and the Humber–South Yorkshire Research Ethics Committee (REC reference: 21/YH/0060) and only consenting individuals were included in the analysis. Our research has been performed in accordance with the Declaration of Helsinki.

## Results

In total, 7.3% of the target population responded to all questionnaires (at 0-, 3-, 6- and 12-months post-infection) and were included in our analytical sample (Fig. 1, Table 1). Compared to the target population, the analytical sample consisted of more females and older CYP (particularly for first-positives). Our sample was also less deprived than the target population and there were regional differences in participation (Table 1).

**Table 1:** Demographics of target population and participants in the analytical sample of the CLoCk Omicron sub-study^β^.

	First positive		Reinfected	
	Target population	Study participants	Target population	Study participants
	(N = 5135)	(N = 345)	(N = 4507)	(N = 360)
% of the target population		6.72%		7.98%
Sex				
Female	2569 (50.03)	202 (58.55)	2217 (49.30)	197 (54.72)
Male	2566 (49.97)	143 (41.45)	2280 (50.70)	163 (45.28)
Age (years)				
11–14	2608 (50.79)	157 (45.51)	3745 (83.10)	301 (83.61)
15–17	2527 (49.21)	188 (54.49)	762 (16.90)	59 (16.39)
Ethnicity	Not available		Not available	
White		279 (80.87)		308 (85.56)
Asian, Asian British		22 (9.57)		27 (7.50)
Mixed		14 (4.06)		19 (5.28)
Black, African, Caribbean		12 (3.48)		4 (1.11)
Other		3 (0.87)		1 (0.28)
Prefer not to say		4 (1.16)		1 (0.28)
Region				
East Midlands	570 (11.10)	36 (10.43)	411 (9.12)	41 (11.39)
East of England	570 (11.10)	37 (10.72)	636 (14.11)	53 (14.72)
London	570 (11.10)	33 (9.57)	361 (8.01)	17 (4.72)
North East England	570 (11.10)	30 (8.70)	296 (6.57)	23 (6.39)
North West England	570 (11.10)	30 (8.70)	499 (11.07)	35 (9.72)
South East England	570 (11.10)	40 (11.59)	889 (19.72)	77 (21.39)
South West England	570 (11.10)	53 (15.36)	350 (7.76)	35 (9.72)
West Midlands	570 (11.10)	41 (11.88)	619 (13.75)	47 (13.06)
Yorkshire and the Humber	570 (11.10)	45 (13.04)	445 (9.87)	32 (8.89)
IMD quintile*				
1 (most deprived)	1200 (23.37)	57 (16.52)	1054 (23.38)	54 (15.00)
2	964 (18.77)	45 (13.04)	800 (17.75)	57 (15.83)
3	928 (18.07)	69 (20.00)	832 (18.46)	61 (16.94)
4	988 (19.24)	78 (22.61)	800 (17.75)	80 (22.20)
5 (least deprived)	1055 (20.55)	96 (27.83)	1022 (22.67)	108 (30.00)

^β^the analytical sample consists of first-positive and reinfected CYP who took part at all data collection sweeps (i.e., 0-, 3-, 6- and 12-months post-testing) *IMD: Index of Multiple Deprivation; calculated from the CYP’s small local area level based geographic hierarchy (lower super output area) at the time of the first questionnaire and used as a proxy for socio-economic status. We report IMD quintiles from most (quintile 1) to least (quintile 5) deprived.

The most common symptoms in first-positive and reinfected CYP at 12-months post-testing were tiredness (35.7 and 33.6% respectively) and sleeping difficulties (27.5% and 28.3% respectively, Table 2). Other common symptoms in both groups included shortness of breath, headaches and a ‘runny nose’ (rhinorrhoea). There was no evidence of difference between the two infection-status groups with respect to total number of symptoms and individual symptoms reported, with the exception of fevers being more common in first-positives (2.6 vs. 0.6% *p* = 0.03) and loss of smell being more common in the reinfected (4.7 vs. 1.7%; *p* = 0.03). Symptom severity and impact were similar in the two infection-status groups. In the 12-month period since infection, 45 first-positives (13.0%) and 74 (20.6%) reinfected CYP spoke to their GP about their Covid infection (*p* = 0.01). In addition, 10 first-positives (2.9%) and 11 (3.1%) reinfected CYP visited a hospital and/or stayed overnight in relation to their Covid infection.

**Table 2:** Reported symptoms N(%), self-rated health, symptom severity and impact, by SARS-CoV-2 status, 12 months post-infection.

	First positive n = 354	Reinfected n = 360	*P* value*
Number of reported symptoms			
0 symptom	162 (46.96)	170 (47.22)	0.84
1 symptom	45 (13.04)	51 (14.17)	
2 symptoms	32 (9.28)	36 (10.00)	
3 symptoms	24 (6.96)	17 (4.72)	
4 symptoms	17 (4.93)	21 (5.83)	
≥ 5 symptoms	65 (18.84)	65 (18.06)	
Specific symptom			
Fever	9 (2.61)	2 (0.56)	0.03
Chills	33 (9.57)	42 (11.67)	0.37
Persistent cough	29 (8.41)	28 (7.78)	0.76
Tiredness	123 (35.65)	121 (33.61)	0.57
Shortness of breath	37 (10.72)	48 (13.33)	0.29
Loss of smell	6 (1.74)	17 (4.72)	0.03
Unusually hoarse voice	8 (2.32)	11 (3.06)	0.55
Unusual chest pain	11 (3.19)	16 (4.44)	0.39
Unusual abdominal pain	8 (2.32)	14 (3.89)	0.23
Diarrhoea	5 (1.45)	8 (2.22)	0.45
Headaches	49 (14.20)	40 (11.11)	0.22
Confusion, disorientation or drowsiness	12 (3.48)	14 (3.89)	0.77
Unusual eye-soreness	11 (3.19)	14 (3.89)	0.62
Skipping meals	31 (8.99)	22 (6.11)	0.15
Dizziness or light-headedness	25 (7.25)	33 (9.17)	0.35
Sore throat	22 (6.38)	22 (6.11)	0.88
Unusual strong muscle pains	5 (1.45)	8 (2.22)	0.45
Earache or ringing in ears	13 (3.77)	13 (3.61)	0.91
Raised welts on skin or swelling	7 (2.03)	2 (0.56)	0.08
Red/purple sores/blisters on feet	2 (0.58)	1 (0.28)	0.54
Sleeping difficulties	95 (27.54)	102 (28.33)	0.81
Low mood	30 (8.70)	27 (7.50)	0.56
Brain fog	24 (6.96)	27 (7.50)	0.78
Concentration difficulties	31 (8.99)	48 (13.33)	0.07
Anxiety	29 (8.41)	27 (7.50)	0.66
Runny nose	44 (12.75)	39 (10.83)	0.43
Sneezing	20 (5.80)	26 (7.22)	0.44
Other	9 (2.61)	14 (3.89)	0.34
Self-rated health^a^	90 (75,95)	90 (80,95)	0.03
Symptom severity^a^	50 (30,60)	50 (30,70)	0.54
Symptom impact^a^	40 (20,70)	40 (10,60)	0.28

*p values from Chi Squared tests; except for self-rated health, symptom severity and symptom impact which were from Mann–Whitney U tests; ^a^Reported as median (IQR), Self-rated health scored on a scale of 0 (worst) to 100 (best); symptom severity from 0 (not severe at all) to 100 (extremely severe) and symptom impact scale from 0 (no impact) to 100 (extreme impact).

**Figure 2:**
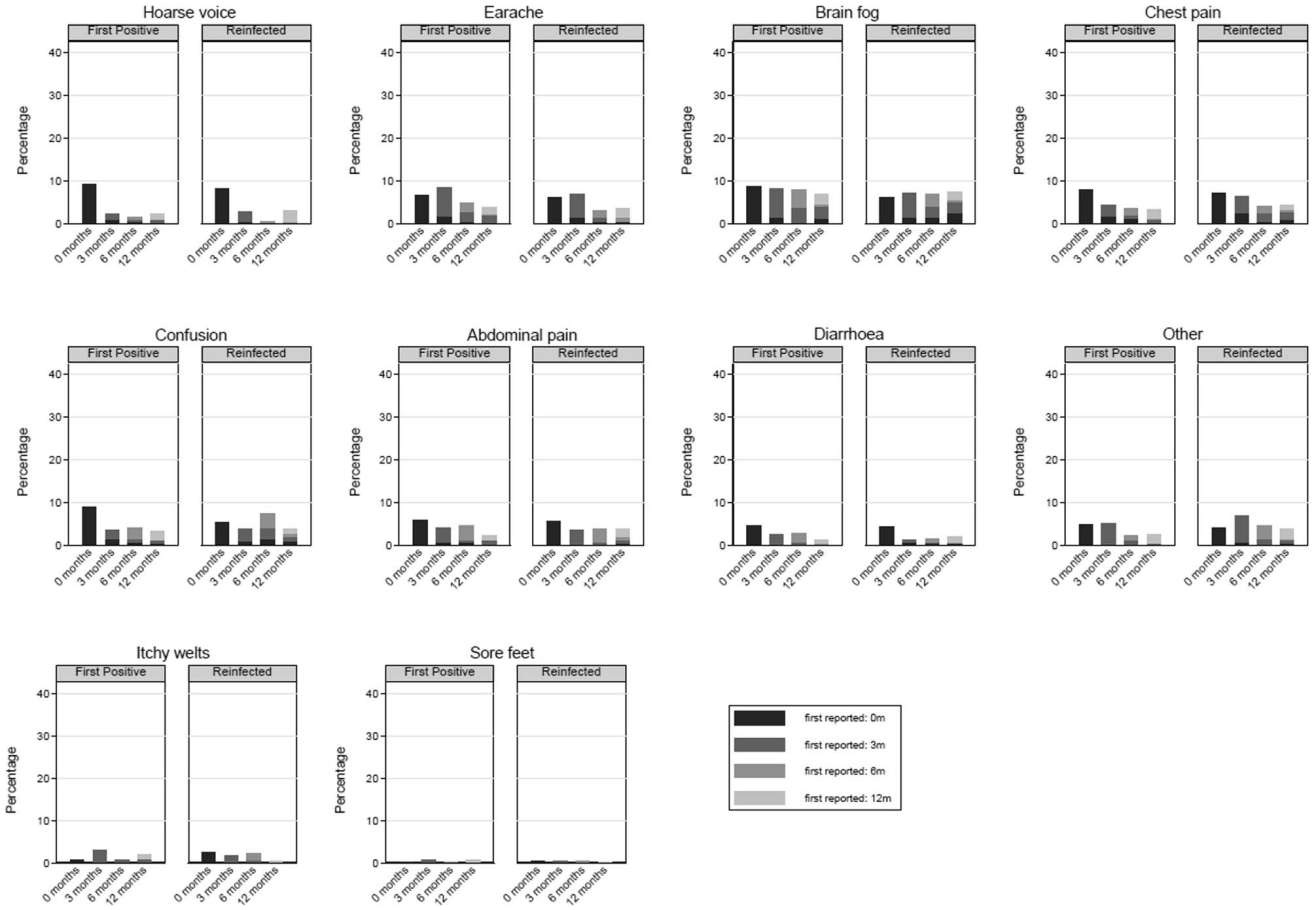
Symptoms with very low overall prevalence (≤ 10%) at all time points.

**Figure 3:**
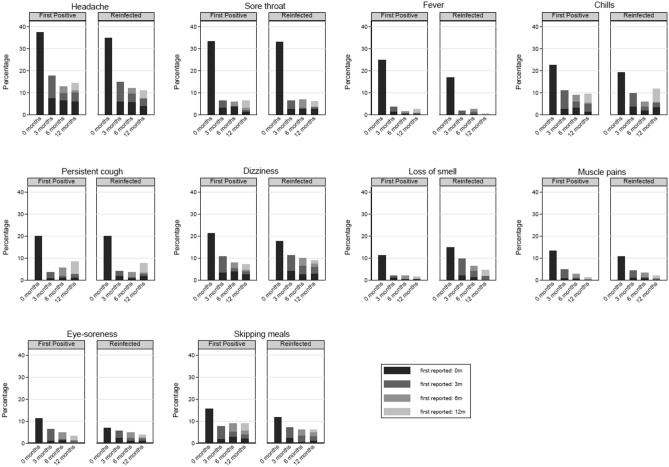
Symptoms where overall prevalence declined from baseline to 12 months post-infection in first-positives and reinfected CYP.

**Figure 4:**
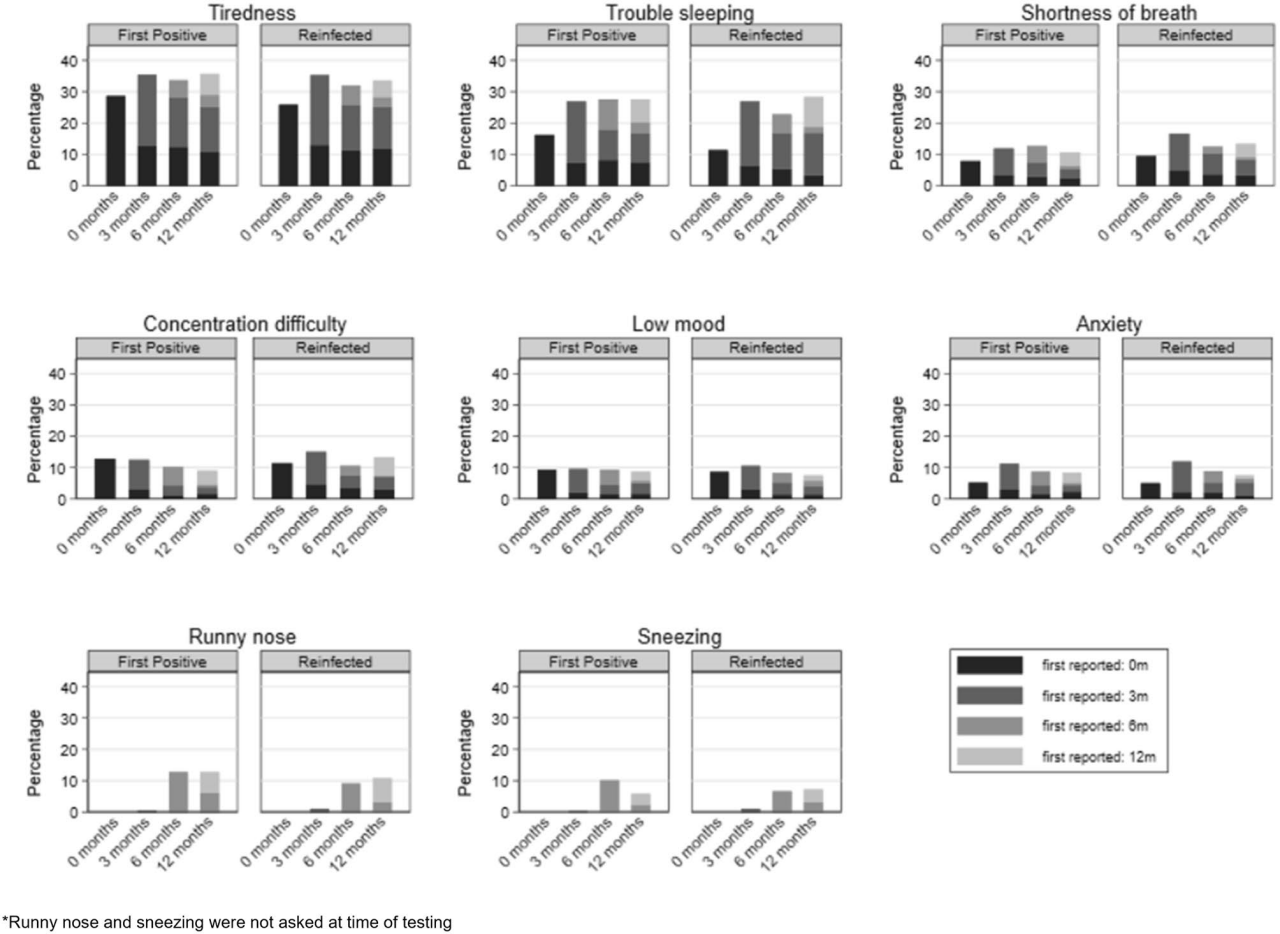
Symptoms where overall prevalence increased or generally stayed high (> 10%) from baseline to 12 months post-infection in first-positives and reinfected CYP*.

**Figure 5:**
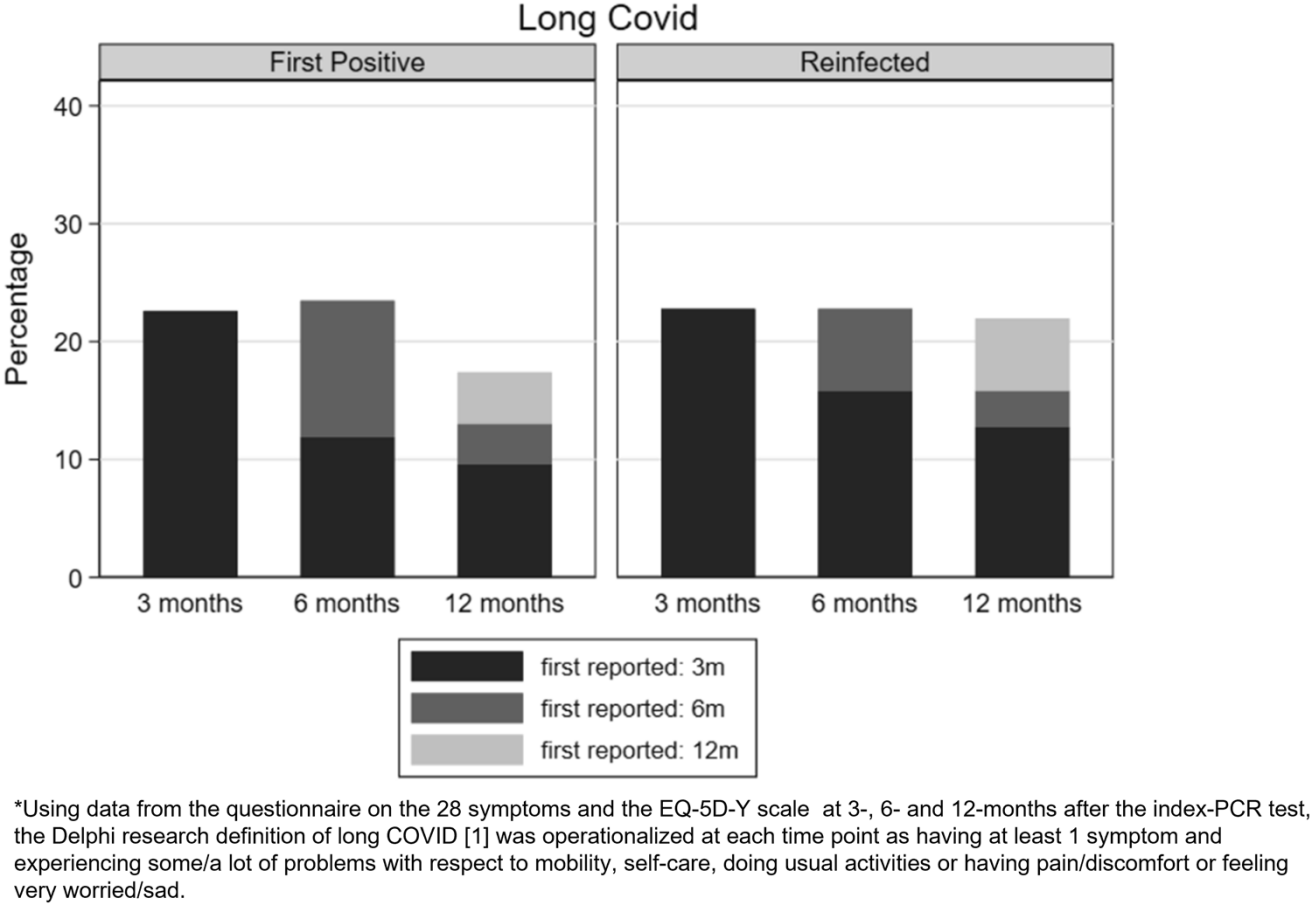
Prevalence of Long Covid* at 3-, 6- and 12-months post-infection by COVID status.

Of the 28 symptoms, 10 (e.g., brain fog, chest pain etc.) had a low prevalence (≤ 10%) at all time-points (Fig. 2). The overall prevalence of 10 other symptoms (e.g., headaches, persistent cough, loss of smell etc.) decreased from time of testing to 12-months post-testing (Fig. 3). For the remaining 8 symptoms (e.g., tiredness and sleeping difficulties), the overall prevalence increased or remained high (> 10%) over the 12-month period (Fig. 4). This is because, while, for example, the prevalence of tiredness being reported by the same CYP declined from time of testing to 3-months and then stayed mostly stable till 12-months, some CYP were reporting tiredness for the first time at 3-, 6- and 12-months post-testing. Therefore, across the two infection status groups, while the within-individual prevalence of most reported symptoms declined over time, the overall prevalence of tiredness, sleeping difficulties, shortness of breath, difficulty concentrating, low mood, anxiety, runny nose and sneezing increased or stayed high over time. Overall, 22.6% of first-positives and 22.8% of reinfected CYP fulfilled our consensus Long Covid definition 3-months post-testing; by 12-months, this was 17.4% in first positives and 21.9% in reinfected CYP (*p* value for difference between infection-status groups in overall prevalence at 12-months = 0.13). In both infection groups, while some CYP meet the Long Covid definition at 6- and 12-months post-infection for the first time, at least half of those meeting the Long Covid definition at 6- and 12-months had met the Long Covid definition at 3-months (Fig. 5). 25 (7.2%) first-positive and 42 (11.7%) reinfected CYP meet the Long Covid definition at all time-points (i.e., 3-, 6- and 12-months post-infection).

By 12 months post-testing, 75.4% of first-positive and 66.7% of reinfected CYP had received at least one dose of a COVID-19 vaccine, with most (~ 48%) having 2 doses (Table 3). There was little difference between vaccination status and number of symptoms, health, quality of life and well-being for either the first-positives or reinfected CYP.

**Table 3:** Characteristics of CYP 12-months post-testing by SARS-CoV-2 and vaccination (by 12 months) status (N(%) or Mean(SD)).

Vaccine dosage	First positive N = 345					Reinfected N = 360				
	0 (n = 85)	1 (n = 47)	2 (n = 163)	3 + (n = 50)	*p* value*	0 (n = 120)	1 (n = 48)	2 (n = 173)	3 + (n = 19)	*p* value*
% in each vaccine group	24.64%	13.62%	47.24%	14.50%		33.33%	13.33%	48.06%	5.28%	
Number of symptoms										
0 symptoms	43 (50.59%)	17 (36.17%)	76 (46.63%)	26 (52%)	0.82	55 (45.83%)	19 (35.58%)	89 (51.45%)	7 (36.84%)	0.23
1 symptom	10 (11.76%)	9 (19.15%)	20 (12.27%)	6 (12%)		15 (12.50%)	4 (8.33%)	30 (17.34%)	2 (10.53%)	
2 symptoms	9 (10.59%)	3 (6.38%)	16 (9.82%)	4 (8%)		13 (10.83%)	6 (12.50%)	16 (9.25%)	1 (5.26%)	
3 symptoms	5 (5.88%)	4 (8.51%)	12 (7.63%)	3 (6%)		6 (5%)	4 (8.33%)	7 (4.05%)	0 (0%)	
4 symptoms	3 (3.53%)	3 (6.38%)	6 (3.68%)	5 (10%)		7 (5.83%)	5 (10.42%)	8 (4.62%)	1 (5.26%)	
≥ 5 symptoms	15 (17.65%)	11 (23.40%)	33 (20.25%)	6 (12%)		24 (20%)	10 (20.83%)	23 (13.29%)	8 (42.11%)	
EQ5DY										
Some/lots of mobility problems	3 (3.53%)	3 (6.38%)	10 (6.13%)	1 (2.00%)	0.58	6 (5.00%)	4 (8.33%)	6 (3.47%)	2 (10.53%)	0.36
Some/lots of self-care problems	2 (2.35%)	5 (10.64%)	7 (4.29%)	1 (2.00%)	0.01	1 (0.83%)	0 (0%)	6 (3.47%)	0 (0%)	0.24
Some/lots of problems with usual activities	6 (7.06%)	7 (14.89%)	14 (8.59%)	2 (4.00%)	0.26	19 (15.83%)	6 (12.50%)	17 (9.83%)	3 (15.79%)	0.47
Some/lots of pain discomfort	8 (9.41%)	7 (14.89%)	22 (13.50%)	4 (8.00%)	0.57	25 (20.83%)	6 (12.50%)	26 (15.03%)	6 (31.58%)	0.17
Very worried, sad/unhappy	2 (2.35%)	3 (6.38%)	12 (7.36%)	1 (2.00%)	0.25	4 (3.33%)	3 (6.25%)	9 (5.20%)	2 (10.53%)	0.55
SDQ										
Total difficulties	10.68 (6.53)	13.68 (7.03)	11.60 (6.55)	11.44 (7.32)	0.12	10.85 (7.02)	12.79 (7.35)	10.47 (6.81)	11.11 (6.40)	0.28
Emotional symptoms	3.35 (2.49)	4.23 (2.78)	3.60 (2.70)	3.76 (2.88)	0.38	3.22 (2.48)	4 (2.78)	3.20 (2.41)	3.16 (2.57)	0.29
Conduct problems	1.67 (1.55)	2.23 (2.06)	1.42 (1.56)	1.70 (2.13)	0.05	1.68 (1.49)	2.27 (1.94)	1.63 (1.81)	1.68 (1.49)	0.10
Hyperactivity/inattention	3.39 (2.55)	4.47 (2.83)	4.07 (2.72)	3.52 (2.41)	0.11	3.98 (2.85)	4.19 (2.77)	3.83 (2.74)	4.16 (2.75)	0.81
Peer relationship problem	2.27 (1.89)	2.74 (1.95)	2.51 (1.94)	2.46 (1.84)	0.53	1.98 (2.04)	2.33 (2.14)	1.82 (1.87)	2.11 (1.52)	0.38
SWEMBS	22.77 (4.78)	20.82 (4.07)	21.45 (4.47)	21.27 (4.95)	0.02	22.14 (4.75)	21.74 (6.04)	22.33 (4.18)	22.69 (4.70)	0.74
Chalder fatigue scale	12.80 (5)	14.00 (5.32)	12.99 (4.99)	13.20 (4.63)	0.19	12.65 (4.75)	12.71 (5.11)	12.51 (4.07)	14.74 (4.28)	0.20
Self-rated health^a^	90 (75, 95)	80 (60, 90)	90 (80, 95)	85 (75, 95)	0.05	90 (80, 95)	85 (70, 95)	90 (80, 95)	80 (70, 95)	0.05
Symptom severity^a^	40 (20, 70)	55 (30, 60)	40 (20, 60)	60 (40, 70)	0.11	60 (40, 70)	50 (30, 60)	40 (30, 60)	20 (10, 60)	0.05
Symptom impact^a^	30 (20, 60)	45 (30, 60)	35 (10, 70)	60 (50, 80)	0.06	50 (10, 60)	50 (20, 70)	30 (10, 50)	20 (10, 30)	0.29

^a^Reported as median(IQR): Self-rated health scored on a scale of 0 (worst) to 100 (best); symptom severity is 0 (not severe at all) to 100 (extremely severe); symptom impact is 0 (no impact) to 100 (extreme impact).

*p values from Chi squared tests for categorical variables and Kruskal–Wallis tests for continuous variables.

SDQ = Strengths and Difficulties Questionnaire; SWEMBS = Short Warwick-Edinburgh Mental Wellbeing Scale. A higher SDQ score indicates more problems; a higher SWEMWBS score indicates better mental well-being; a higher fatigue score is more severe.

## Discussion

The findings from this study largely aligned with our hypotheses. Commonly experienced symptoms included tiredness, sleep difficulties, headache and shortness of breath. Although it was largely true that the overall prevalence of symptoms at 12-months was lower compared to baseline, as well as 3- and 6-months post-testing, indicating that symptoms after Covid-19 infection do improve over time, there were some notable exceptions. For example, within-individuals, the prevalence of tiredness and sleeping difficulties initially declined over time but then plateaued, indicating that some CYP continued to experience these symptoms over the course of the follow-up period. We found that symptom profiles of the first-positive and reinfected groups were broadly similar at 12-months post-testing, consistent with our initial hypothesis. Those reinfected were significantly more likely than the first-positive group to contact their GP about their SARS-CoV-2 infection. However, the two groups were similar in terms of symptom severity and impact according to self-report. Finally, symptom profiles did not differ by vaccination status.

Taking the findings together, we are able to draw some conclusions regarding what happens to CYP 12-months after infection with the Omicron variant in January 2022 in terms of the frequency, pattern and severity of persisting symptoms. In this study, about half the cohort reported at least one symptom 12-months post-infection, with no difference in prevalence between first-positive and reinfected CYP. The most common symptoms were tiredness and sleeping difficulties; more specific symptoms such as loss of smell were much less common affecting 2–5% of the cohort. Overall, by 12-months, 17–22% of first positive and reinfected CYP met our research consensus definition of Long Covid^1^, and participants in both groups typically reported their symptoms to be of moderate severity and impact. These figures need to be considered in light of the high level of tiredness and sleeping difficulties in the general population of adolescents. For example, one pre-pandemic survey performed from 2002 to 2004 found that 21% of adolescent girls and 7% of adolescent boys in the general population were severely fatigued^20^. Consequently, it is important to emphasise that the focus of our study was to describe symptom profiles 12-months post-Omicron infection and not on determining causality of reported symptoms in relation to the Omicron infection. Nonetheless, our findings raise important questions regarding the attribution of self-reported symptoms up to 12-months after acute SARS-CoV-2 infection. These findings also highlight the limitations of current long COVID clinical case^6^ and research definitions^1^, emphasising a need for more detailed investigations and identifying a reliable and objective biomarker for long Covid. Notably, while the prevalence of symptoms at 12-months post-infection appears high, only 3% of these CYP (n = 21) visited a hospital and/or stayed overnight in relation to their SARS-CoV-2 infection over the 12-month post-infection period.

Compared to other variants, for example, when the wild type and alpha variants were dominant, symptom prevalence appears to be lower after Omicron infection. In the main CLoCk study, 12-months post index-test, 61–74% with at least one positive SARS-CoV-2 test had symptoms at 12-months^21^. However, when looking at 5+ symptoms at 12-months, the proportion of those infected with the Omicron variant and wild type/alpha were similar. 17–22% of test positives infected with Omicron and 27% of test positives infected with wild type/alpha met our published research consensus definition of post-Covid-19 condition^1^. The pattern of symptoms was also similar, regardless of the variant with tiredness, sleeping difficulties, shortness of breath and headaches dominating. For both variants, symptom profiles, mental health, wellbeing, fatigue and quality of life generally did not vary substantially by vaccination status. In summary, the reported symptom prevalence, pattern and impact appear to be similar across the Omicron and Alpha/wild type variants up to 12 months post-infection.

In a complex landscape of Long Covid research, these findings provide important data on the impact of different variants on Long Covid up to 12 months post-infection. The lack of any substantial difference in the prevalence, or impact of reported symptoms between variants, irrespective of Covid-19 vaccination, means that services treating Long Covid 12-months post infection can focus on providing a uniform service that addresses the heterogeneity in presentations regardless of the time (and, therefore, strain) of infection. Importantly, too, we need additional studies comparing post-infection symptoms in CYP exposed to different viruses such as RSV or influenza, for example, to truly understand the risk of long Covid after SARS-CoV-2 infection. It is likely that for some children, the reported symptoms will have a significant impact on their quality of life and their ability to perform daily activities. Any attempt to establish Long Covid clinics for CYP should, therefore, focus on evaluating and supporting CYP with debilitating symptoms rather than their history of any previous (or repeated) SARS-CoV-2 infection. This is particularly important in countries, such as the UK, that have stopped providing or requiring routine community SARS-CoV-2 testing. While the symptom profile is very similar across variants, restrictions imposed on CYP in England varied over time. For example, unlike the wildtype wave, schools largely remained open during the Omicron wave^22^ and there were fewer national restrictions compared to previous waves of infections. This suggests that a large proportion of CYP were infected and/or reinfected with the Omicron variant. It is, therefore, noteworthy that the vast majority of CYP have recovered without complication and that the reported symptom profiles post-infection have been consistently similar throughout the course of the Covid-19 pandemic.

As with the main CLoCk study^21^, it appears that some CYP newly report Long COVID symptoms at 12 months, and some report symptoms consistently at each time point. The WHO definition of Long Covid in young people requires that symptoms first arise within 3 months of infection^6^ and, as such, those with newly reported symptoms over 3 months post-infection, would not be considered to have Long Covid. The difference between clinical and research definitions highlights the importance of a universally accepted definition of Long Covid for the paediatric population.

This study has some limitations. First, there is no negative control group. Given that 99.3% of 12–18-year-olds had SARS-CoV-2 antibodies^8^, the relevant comparison group needs to be determined in relation to the hypotheses. For this study, the most relevant comparison is the data from the main CLoCk study that has an almost identical methodology but assessed the impact of infection with another variant. Second, we do not have any information on additional infections since April 2022 when all community testing for SARS-CoV-2 was stopped. Additionally, although respondents in the main CLoCk study are broadly representative of the target population^23^, only 7.3% of those approached for this smaller Omicron sub-study participated at all time points. However, respondents to the Omicron sub-study are broadly similar to the target population, albeit with some differences (e.g., by region). Third, non-responders may be more likely to experience symptoms which could reduce their likelihood of participation. This would lead to an under-estimation in the prevalence figures reported. Equally, they could be less invested in being involved in a study than young people still experiencing symptoms. Overall, the participants in the main CLoCk study are largely representative of the target population^23^.

Despite these limitations, this study largely achieves its goal of contributing to our understanding of persistent symptoms after initial and repeated SARS-CoV-2 infection with different variants in CYP. We conclude that the symptom profile and impact at 12 months after primary infection and re-infection with the Omicron variant is largely similar to those of other variants. The changes in prevalence of meeting the Long Covid criteria varied during the 12-month study period, which emphasises the need for longitudinal assessment of children generally for these symptoms and consideration for longer term effects. Nonetheless, our findings suggest that understanding the time of infection and therefore, type of variant, may not be a noteworthy factor when developing investigation and management plans. Further studies are needed to assess causality of reported symptoms up to 12-months after SARS-CoV-2 infection, with less attention given to variant strain.

## Data Availability

Data is not publicly available. All requests for data will be reviewed by the Children & young people with Long Covid (CLoCk) study team, to verify whether the request is subject to any intellectual property or confidentiality obligations. Requests for access to the participant-level data from this study can be submitted via email to ich.clock@ucl.ac.uk with detailed proposals for approval. A signed data access agreement with the CLoCk team is required before accessing shared data.
